# Antipsychotic Discontinuation through the Lens of Epistemic Injustice

**DOI:** 10.1007/s10597-024-01274-7

**Published:** 2024-04-08

**Authors:** Helene Speyer, Lene Falgaard Eplov, David Roe

**Affiliations:** 1 Mental Health Center Copenhagen, Copenhagen University Hospital, Gentofte Hospitalsvej 15, 4., Hellerup, 2900 Denmark; 2 Department of Community Mental Health, University of Haifa, Haifa, 2900 Israel

In an era in which progress in mental health care has increased awareness about recovery, autonomy and human rights, clients may be more open about their decisions to go “against medical advice”. This could generate a new type of clinical encounter in which clinicians must consider additional legal and moral issues and client rights. In this essay, we argue that *epistemic injustice* is a useful concept to understand and discuss situations in which clients and medical professionals disagree about decisions to stop or maintain treatment with antipsychotic medication.

After decades of research to improve adherence to antipsychotic treatment for people with schizophrenia, papers and debates questioning the risk-benefit ratio of maintenance treatment are now emerging (Correll et al., 2018; Davidson, 2018; Gupta, Cahill et al., 2018a; Gupta et al., 2018b; Moncrieff, 2015; Murray et al., 2016; Speyer & Roe, 2024; Steingard, 2018) Taking antipsychotic medication can, on average, reduce the risk of a psychotic relapse from approximately 64–27% within the first year, as reported in a recent meta-analysis of available randomized controlled trials(Leucht et al., 2012). This reduction signifies a substantial effect size compared to numerous medical interventions.

Despite this, a noteworthy trend persists: Approximately 90% of individuals diagnosed with schizophrenia attempt to discontinue medication within the initial years (Stürup et al., 2022). While this phenomenon has been recognized for decades, the prevailing strategy to address it has revolved around developing interventions to enhance adherence. With our contemporary focus on client rights and recovery, a shift is taking place from expert recommendations to embracing shared decision-making, incorporating the client’s personal values and preferences(Mccormack et al., 2018). With this shift, psychiatry must be equipped for another type of dialogue in clinical consultations.

There are valid reasons to consider discontinuing medication. First, approximately one-third of those diagnosed with schizophrenia can cease medication without experiencing a relapse(Gotfredsen et al., 2019). Second, around 20% fall under the category of “treatment-resistant”, experiencing no symptom alleviation but encountering side effects. Last, some individuals may opt to endure psychotic symptoms rather than contend with medication-induced side effects. However, discontinuation of antipsychotic medication may be linked to severe relapses, hospitalizations, a heightened risk of harmful behavior, and the development of treatment resistance. Balancing risks and benefits are an inherently complex and emotionally charged task that cannot be resolved by following the sometimes binary recommendations of clinical guidelines.

Challenging the legitimacy of antipsychotic long-term treatment is not only an academic issue, but also a real-life dilemma of health care professionals working with people with psychosis, and therefore ethical reflections are a necessity. In this article, we suggest that the concept of epistemic injustice (Fricker, 2007) can add a useful perspective to the academic debate as well as to the ethical considerations in clinical encounters.

## Epistemic Injustice

Epistemic injustice, a concept introduced by feminist philosopher Miranda Fricker, delineates the mistreatment inflicted upon individuals in their roles as knowers or conveyors of knowledge (Fricker, 2007). The term “epistemic” pertains to knowledge, and injustice manifests when someone is unfairly judged as an unreliable source of information due to unjustified prejudices. Within psychiatry, individuals diagnosed with mental illnesses may face diminished credibility, stemming from *unjustified negative preconceptions* about their capacity to provide reliable knowledge. It is essential to acknowledge that certain individuals, under specific circumstances, may indeed struggle to deliver trustworthy information due to various factors such as delusions. However, the primary focus of this paper lies in highlighting the unwarranted negative preconceptions.

Fricker posits that epistemic injustice can be further categorized into *testimonial injustice* and *hermeneutical injustice.* Testimonial injustice occurs when a statement is accorded lower credibility based on unjustified preconceptions, such as negative stereotypes. Hermeneutical injustice occurs when testimonial injustice influences the inclusion of certain types of knowledge in our collective understanding. Among other problems, this constrains an individual’s ability to make sense of their experiences.

The concept of injustice requires that the delegitimization of testimonies is due to *unjustified prejudices*. Unjustified biases against individuals with serious mental illnesses encompass negative stereotypes, such as perceiving individuals labeled with schizophrenia as dangerous per se, having poor “insight” into their own symptoms and need for treatment, and suffering from a chronic, deteriorating course. Letting these unwarranted stereotypes influence the assessment of a decision to discontinue medication within the shared decision-making process, gives rise to a situation of epistemic injustice—a wrongdoing committed against someone functioning as a transmitter of knowledge.

## Testimonial Injustice

When a person diagnosed with schizophrenia expresses a wish to discontinue medication, their perspective is given less credibility than people without mental health problems, as they are perceived as lacking insight into their own best choice. Adherence to antipsychotic medication among people with serious mental illness is quite widely recommended by official guidelines on antipsychotic medication(Correll et al., 2022). However, most people make at least one attempt to reduce their dosage or stop medication altogether(Stürup et al., 2022). For many, stopping medication is an active decision, as anticipated benefits are outweighed by risks and adverse effects. Often, health care professionals are reluctant to support this decision because it is not in line with their clinical judgment and guidelines(Roed et al., 2023). Medical professionals’ reluctance to discuss or support tapering leaves clients alone, discontinuing medication without support and observation. Paradoxically, this may leave them at higher risk of relapse, while clinicians can deny responsibility by stating that the decision was made “against medical advice”. There is no easy answer for these situations.

The perspective that epistemic injustice can offer is an honest reflection on why clinicians often are reluctant to support a person who asks for help to taper antipsychotic medication. If the answer is unjustified preconceptions, then reluctance to respect the choice of the person may be a case of epistemic injustice. Historically, disagreements between patients and clinicians have been (Lysaker et al., 2007, 2009)conceptualized as “lack of insight” but this has long been challenged (Lysaker et al., 2007, 2009) and viewpoint is currently contested (Slade & Sweeney, 2020).

The concept of insight is often vaguely conceptualized and has been used in cases in which clients disagree with medical professionals about diagnoses and the benefits of treatment. When vaguely applied, “lack of insight” allows the epistemic devaluing of and testimonial injustice against anyone who offers an alternative explanation for their condition or who chooses to stop taking their medication. This conflicts with contemporary developments in mental health that give epistemic priority to the voices of service users, who are encouraged to develop narrative insight(Roe et al., 2008). When these developments are not followed, a person may have full insight, but perceptions about the causes of their struggles or how to best deal with them that are not in line with those of the treating psychiatrist can result in reducing their credibility as *knowers*.

The idea of epistemic injustice encourages clinicians to think in more nuanced ways and ask themselves if reluctance to support and supervise people during tapering could be based on unjustified negative stereotypes such as dangerousness, preconceptions about what a good life is like, or bias about chronicity. Another obscuring issue may be tensions between clients and medical professionals in willingness to take risks(Speyer & Roe, 2024; Zisman-Ilani et al., 2021). While running the risk of a relapse may be an important step in the path of personal recovery from a client’s perspective, being the treating clinician in a process that does not follow guidelines and could lead to clinical worsening may pose legal as well as moral issues.

## Hermeneutical Injustice

Hermeneutical injustice unfolds when testimonial injustice, marked by a failure to appreciate individuals’ expressed desires to cease medication, influences the types of knowledge incorporated into a collective pool of information. In the context of antipsychotic discontinuation, this collective knowledge pool exhibits some notable gaps. These include (1) potentially severe consequences of long-term treatment, (2) severity and frequency of withdrawal symptoms, and (3) development of the safest possible tapering strategies.

The literature suggests that long-term treatment with antipsychotic medication may cause brain shrinkage or dysregulation of dopamine receptors, which may increase cognitive dysfunction and risk of iatrogenic psychotic symptoms(Goff et al., 2017). These hypotheses are neither confirmed nor rejected and receive surprisingly limited attention.

Clinical guidelines currently lack guidance and support regarding the safe tapering of medication that mitigates withdrawal symptoms and reduces the risk of relapse. The need for safe tapering approaches, including tools to identify withdrawal symptoms, has persisted since the inception of antipsychotic agents but has largely been overlooked by the scientific community(Read, 2022), in favor of prioritizing strategies to improve adherence. Global, user-led initiatives have led to online communities supporting each other during tapering of medication(Framer, 2021). They serve as rich sources of knowledge about withdrawal symptoms and methods to avoid them by altering pharmaceutical products to take smaller doses. The ignorance of the scientific community of these patient needs(Cooper et al., 2020) can be seen as a case of hermeneutic injustice, in which this knowledge about withdrawal symptoms is not described in textbooks, neglecting to acknowledge that many patients have experienced them as problematic. In the worst case, doctors may unknowingly misinterpret withdrawal symptoms as signs of recurring, underlying disease processes(Guy et al., 2020). We do not claim that the existence of underlying disease is never the case but we suggest that it may not always be so. Horowitz et al. developed a biologically plausible tapering strategy(Horowitz et al., 2021)building on the hypothesis that slow, hyperbolic dose reduction minimizes the risk of withdrawal symptoms. However, no trial has compared tapering strategies head-to-head, a deficiency that seems striking compared to the number of studies examining medication initiation.

This condition of injustice arises from a biased research priority that favors medication adherence and maintenance, neglecting the reality that most people attempt to discontinue at least once and need information and support to do so as safely as possible. The skewed priority may be influenced by the prominent status of the medical model and negative stereotypes surrounding the chronicity of severe mental illnesses. The academic community’s skepticism about withdrawal symptoms can be viewed as a case of structural testimonial injustice, as user-driven research has highlighted for some time(Framer, 2021).

## Conclusion

Decisions about long-term treatment with antipsychotic medication remain complex and emotionally charged, especially with the current priority on client rights, autonomy and shared decision-making. We argue here that the current debate about risks and benefits associated with antipsychotic medication can be fruitfully analyzed through the lens of epistemic injustice.

First, we point out that there can be good reasons to discontinue medication, and as a point of departure, that people diagnosed with psychosis should be seen as credible knowers when they express such a wish. Delegitimizing these people as less able to deliver trustworthy testimonies, based on negative prejudices such as “poor insight”, is not aligned with contemporary values in recovery-oriented mental health and can be seen as a case of testimonial injustice.

Further, we point out a number of unanswered research questions that need to be addressed, including the need to develop safer possible ways to identify those who do not need medication without jeopardizing the health of those who do, making sufficiently small doses available so people don’t need to alter pills, and finally, to educate doctors in differentiating between relapses and withdrawal symptoms. The limited knowledge about safe tapering processes reflects a skewed professional prioritization and older views of control and paternalism, rather than shared decision-making. The scarcity of research on the reasons and methods behind individual choices to discontinue antipsychotic medication, leading to the absence of clinical guidelines, can be interpreted as an indication of hermeneutical injustice.

In conclusion, we argue that both sides of the medication discontinuation debate should approach questions about medication with epistemic humility. There are no clear right or wrong answers and people should be given the opportunity to make their own choices on their personal path to recovery, whether this involves choices to risk relapse or long-term medication.
